# Rescuing zinc anode–electrolyte interface: mechanisms, theoretical simulations and *in situ* characterizations

**DOI:** 10.1039/d4sc00711e

**Published:** 2024-04-08

**Authors:** Zhenjie Liu, Xiaofeng Zhang, Zhiming Liu, Yue Jiang, Dianlun Wu, Yang Huang, Zhe Hu

**Affiliations:** a Guangdong Provincial Key Laboratory of New Energy Materials Service Safety, College of Materials Science and Engineering, Shenzhen University Shenzhen 518055 Guangdong P. R. China huzhe@szu.edu.cn; b The Hong Kong University of Science and Technology (Guangzhou), Advanced Materials Thrust Nansha Guangzhou 511400 Guangdong P. R. China yanghuang@hkust-gz.edu.cn

## Abstract

The research interest in aqueous zinc-ion batteries (AZIBs) has been surging due to the advantages of safety, abundance, and high electrochemical performance. However, some technique issues, such as dendrites, hydrogen evolution reaction, and corrosion, severely prohibit the development of AZIBs in practical utilizations. The underlying mechanisms regarding electrochemical performance deterioration and structure degradation are too complex to understand, especially when it comes to zinc metal anode–electrolyte interface. Recently, theoretical simulations and *in situ* characterizations have played a crucial role in AZIBs and are exploited to guide the research on electrolyte engineering and solid electrolyte interphase. Herein, we present a comprehensive review of the current state of the fundamental mechanisms involved in the zinc plating/stripping process and underscore the importance of theoretical simulations and *in situ* characterizations in mechanism research. Finally, we summarize the challenges and opportunities for AZIBs in practical applications, especially as a stationary energy storage and conversion device in a smart grid.

## Abstract 2

The protective mechanisms, theoretical simulations and *in situ* characterizations of zinc metal anode–electrolyte interface are critically analyzed, and the possible development directions are emphasized.

## Graphical abstract

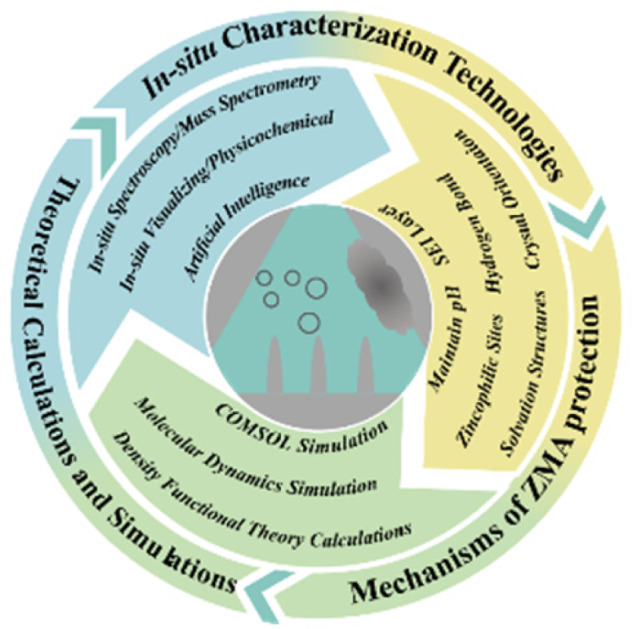

## 1. Introduction

Since Kang *et al.* first proposed the concept of aqueous zinc-ion batteries (AZIBs),^1^ AZIBs have received tremendous attention due to their high safety, abundance, and satisfactory performance. Besides, the development of AZIBs promotes the studies on other aqueous batteries (*e.g.*, Zn–I_2_ batteries), which share similar mechanism and cell configuration.^2–4^ However, currently, AZIBs still encounter many challenges that hinder their further development as a practical alternative to commercial batteries (*e.g.*, Li-ion batteries). Although researchers have made great efforts to improve the electrochemical performance, some tough issues remain, including the co-intercalation of proton and Zn^2+^, dissolution of cathode materials, sluggish kinetics during charge and discharge, and unstable Zn metal anode (ZMA).^5,6^ Among these challenges, the ZMA is the most serious one, resulting in the quick capacity decay of AZIBs in their long-term service.

**Fig. 1:**
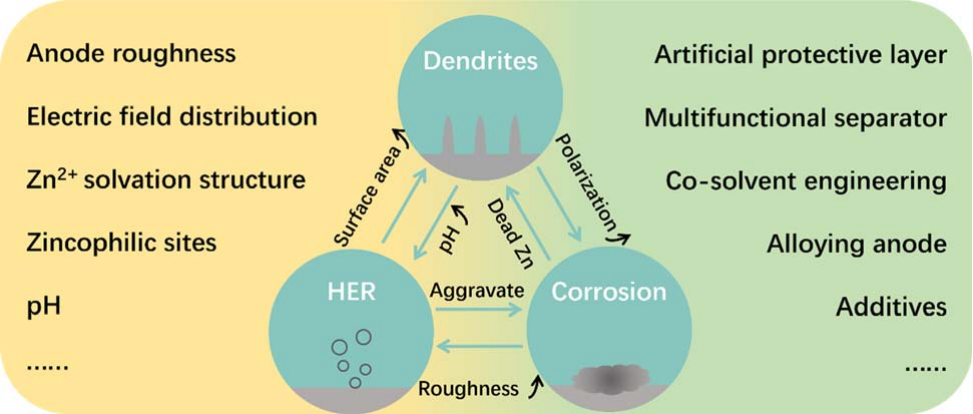
The issues, influencing factors and modification strategies of the ZMA–electrolyte interface.

In the aqueous electrolyte, ZMA will undergo a chemical conversion reaction between Zn atoms and Zn^2+^, *i.e.*, the plating/stripping of ZMA, during the discharge and charge processes. It is generally accepted that the problems occurring on the ZMA–electrolyte interface are the main reasons for ZMA's instability. For example, the nucleation and growth of Zn^2+^ on the ZMA surface during Zn plating is usually accompanied with the growth of irregular Zn dendrites, which will penetrate the separator membrane and eventually cause the short circuit in AZIBs. In addition, the side reactions taking place at the ZMA–electrolyte interface during the plating/stripping (*e.g.*, hydrogen evolution reaction (HER) and corrosion) can cause low coulombic efficiency (CE) and low capacity, which considerably degrades the cycling stability of ZMA. In fact, the issues happening at the ZMA–electrolyte interface are interdependent and entangled with each other, as shown in Fig. 1, which would break the balance of the ZMA–electrolyte interface and finally lead to the failure of ZMA and AZIBs.^7,8^

When addressing the instability issues of ZMA, it requires a comprehensive understanding of the underlying mechanisms and influencing factors that are related to the ZMA–electrolyte interface. For instance, during Zn plating, the zinc ions near the ZMA–electrolyte interface will undergo a series of reaction steps, such as migration, desolvation, diffusion, and reduction at the nucleation sites. Due to the different electric field and Zn^2+^ distribution, Zn atoms prefer to accumulate and continuously grow at some protuberances of the ZMA surface, which results in Zn dendrites after repeated Zn plating. Therefore, the Zn plating result is intricately connected to the distribution of electric field, concentration of Zn^2+^, and amount of zincophilic sites, all of which could be influenced by the roughness of the ZMA–electrolyte interface.^9–11^ Moreover, Zn(H_2_O)_6_^2+^, as the primary solvation structure in the aqueous electrolyte, is considered as a crucial factor that induces HER at the ZMA–electrolyte interface. The occurrence of HER would then lead to a localized pH elevation at the ZMA–electrolyte interface, thereby exacerbating the corrosion reaction at the surface of ZMA.^12^ Based on the above understanding, it is apparent that the stabilization of the ZMA–electrolyte interface, determined by interface roughness, Zn^2+^ solvation structure, and electrolyte pH, is the key for solving the problems of unstable ZMA in the aqueous electrolyte. Accordingly, the improvements of the ZMA–electrolyte interface should be conducive to enhancing the electrochemical performance of AZIBs in theory.^13,14^

Currently, many strategies have been developed to enhance the properties of the ZMA–electrolyte interface, for example, optimizing the crystalline structure of ZMA, constructing artificial interface on ZMA,^15,16^ designing multifunctional separator and current collector,^17–19^ and regulating the electrolyte compositions.^20–23^ To further improve these strategies for better AZIBs, it is important to uncover the relationships between interface structure and battery performance.^24,25^ There are many characterization methods that have been applied to investigate the structure of the ZMA–electrolyte interface. However, these methods are mostly *ex situ* techniques, which fail to provide continuous information to clearly illustrate the dynamic evolution of the ZMA–electrolyte interface. Such a lack of information of the interface structure from *ex situ* characterization might be less effective in guiding future research on the ZMA–electrolyte interface. Thus, *in situ* characterization methods that can provide abundant information of the battery during any period of charge and discharge are indispensable for explaining the structure and performance relationship of the ZMA–electrolyte interface. Moreover, as a supplementary technique for *in situ* characterizations, theoretical simulations provide detailed information about the structure change of the ZMA–electrolyte interface at atomic and electronic levels under specified conditions. Apparently, the combination of *in situ* characterizations and theoretical simulations should be an ideal scheme for understanding how to improve the ZMA–electrolyte interface. However, only a few studies summarize the important roles of *in situ* characterizations and theoretical simulations in the research on the ZMA–electrolyte interface.

To fill the gap of current studies, we focus on discussing three objects in this review, *i.e.*, ZMA–electrolyte interface protective mechanisms, theoretical simulations, and *in situ* characterizations. At first, we systematically overview six protective mechanisms for enhancing the stability of the ZMA–electrolyte interface, including the provision of zincophilic sites, regulation of crystal orientation, modulation of solvation structure, reconstitution of hydrogen bond network, maintenance of pH, and formation of the solid electrolyte interphase (SEI) layer. Subsequently, we have discussed how common theoretical simulations and various advanced *in situ* characterizations are used to study the reaction kinetics of Zn plating/stripping and the dynamic changes at ZMA–electrolyte interface, and ultimately evaluate various optimization strategies and their influence on the battery performance. At the end of this review, we will provide our perspectives and insights into the future development of the ZMA–electrolyte interface and the *in situ* characterizations. We believe that our review will be helpful for designing the highly stable ZMAs for high-performance AZIBs.

## 2. Electrolyte engineering-induced protective mechanisms

**Fig. 2:**
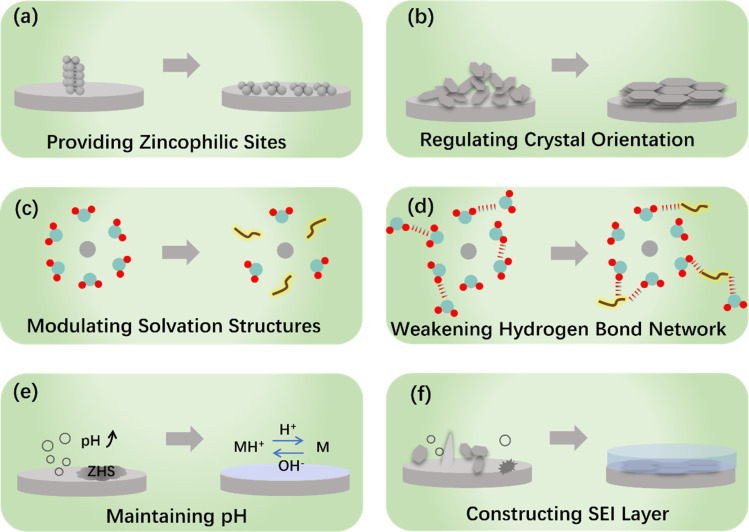
Protective mechanism of electrolyte engineering for ZMA.

In recent years, there has been a significant increase in research investigating various protection strategies that aim at enhancing the stability of the ZMA–electrolyte interface. Taking into account the simplicity and efficiency in controlling the electrolyte compositions, we primarily focus on summarizing the *in situ* electrolyte engineering strategies. Hence, the coverage of other protection strategies in this section is not exhaustive. More detailed information of other protection strategies can be obtained from ref. 26 and 27. The commonly used electrolyte engineering strategies can be classified into the following categories: electrolyte additives,^28–30^ co-solvent engineering,^31–34^ gel-electrolytes,^35,36^ deep eutectic electrolytes,^37–39^ and high concentration salts^22,40–43^ This section, as depicted in Fig. 2, presents a review of the mechanisms involved in electrolyte engineering, including the provision of more zincophilic sites, regulation of crystal orientation, modulation of solvation structure, weakening of the hydrogen bond network, maintenance of pH, and construction of the SEI layer.

Table 1 systematically summarizes the electrolyte composition and electrochemical properties of ZMA under different mechanisms. However, due to the lack of evaluation standard, the underlying mechanisms for each additive remain unclear. Currently, the main issues of additive strategies are the high additive dosage, low current density and shallow discharge depth, which fail to meet the application requirements. Moreover, the impacts of additive on the electrolyte stability and cathode compatibility are sometimes overlooked. Therefore, further studies are required to understand the ZMA–electrolyte interface, which mainly include the design and selection of electrolyte additive, the structure–function relationship between the additive/interface components and electrochemical properties, as well as the investigation of the dynamic performance and electrochemical behavior of ZMA.

**Table 1:** Comparison of performance reported for different protective mechanisms *via* electrolyte engineering

Protective mechanisms	Electrolyte/anode	Cycling performance [h (mA cm^−2^, mA h cm^−2^)]	Reference
Providing zincophilic sites	0.2 wt% sericin/2 M ZnSO_4_ (ZS)	2860 (1, 2)	45
	10 mM α-cyclodextrin (CD)/3 M ZS	200 (5, 5)	46
	10 mM Ce_2_(SO_4_)_3_/1 M ZS	700 (5, 1)	47
	8.5 mM La(NO_3_)_3_/2 M ZS	1200 (1, 1)	48
	4 mM NiSO_4_/2 M ZS	900 (1, 1)	49
Regulating crystal orientation	20 mM BMIm^+^/2 M ZS	1400 (5, 5)	62
	500 mM sorbitol/1 M ZS	480 (5, 5)	63
	β-CD/2 M ZS	1700 (4, 2)	65
	5 vol% DX/2 M ZS	1000 (5, 5)	69
	Cys-Zn@Zn	2000 (2, 2)	70
Modulating solvation structure	0.5 wt% silk fibroin/1 M ZS	>1600 (1, 1)	77
	1 M triethylmethyl-ammonium chloride/0.5 M ZnCl_2_	500 (5, 2.5)	78
	0.05 M ethylene diamine tetraacetic acid/1 M ZS	3000 (5, 1)	81
	4 M 1-ethyl-3-methylimidazolium chloride/2 M ZS	500 (1, 1)	83
	40 vol% methanol/5 mol kg^−1^ Zn(BF_4_)_2_/ethylene glycol	1600 (2, 1)	87
Weakening hydrogen bond network	5 mM trehalose/1 M ZS	1300 (5, 2.5)	89
	100 mM xylitol/2 M ZS	1000 (5, 1)	90
	2 mg mL^−1^ CH_6_NPO_3_/2 M ZS	900 (5, 2.5)	91
	DMF-50% + PEG-30%/2 M Zn(OTF)_2_	1000 (2, 2)	92
	4 mol kg^−1^ Zn(BF_4_)_2_ in ethylene glycol	4000 (0.5, 0.25)	94
	2 M Zn(OTF)_2_ in 2H1D (volume ratio of H_2_O : DMF)	>1000 (50, 50)	95
Maintaining pH	2 M LiCl/3 M ZS	170 (0.2, 0.03)	98
	Acetic acid/tetramethylene sulfone/3 M Zn(OTF)_2_	300 (5, 5)	99
	75 mM Na_4_EDTA/2 M ZS	2000 (5, 2)	103
	0.025 M NH_4_OAc/2 M ZS	3500 (1, 1)	104
	40 vol% γ-butyrolactone/1.6 mol kg^−1^ ZnCl_2_	600 (10, 2)	109
Constructing SEI layer	20 mM Zn(NO_3_)_2_/3 M Zn(OTF)_2_	1200 (0.5, 0.5)	119
	1% fluoroethylene carbonate/2 M ZS	1000 (4, 1)	120
	0.05 mM sulfanilamide/2 M ZS	4800 (2, 2)	123
	25 mM Zn(H_2_PO_4_)_2_/1 M Zn(OTF)_2_	220 (5, 1)	125
	1.3 M ZnCl_2_–H_2_O–DMSO	1000 (0.5, 0.5)	127

### 2.1. Providing zincophilic sites

The operation of Zn nucleation involves the migration, desolvation, and diffusion of Zn^2+^, and the diffusion process precisely depends on the zincophilic sites. The dense zincophilic sites favor achieving a constant 3D diffusion process, thereby guiding the subsequent uniform plating of Zn. However, the rampant 2D diffusion process occurring at the ZMA–electrolyte interface would lead to the production of Zn dendrites. For instance, Zhang *et al.*^44^ proposed that the polyacrylamide additive with abundant acyl groups serves as a guiding intermediary to provide more nucleation sites. A uniform electric field and charge distribution are discovered, resulting in a smooth ZMA surface during cycling. Wang *et al.*^45^ selected sericin molecules as electrolyte additives, which contain zincophilic functional groups. The proposed electrolyte additive adsorbs on the ZMA surface, resulting in denser ZMA after Zn plating. Zhao *et al.*^46^ took advantage of the hydrophobic internal cavity and hydrophilic external surface of cyclodextrins to manipulate and boost the kinetics and stability of ZMA.

**Fig. 3:**
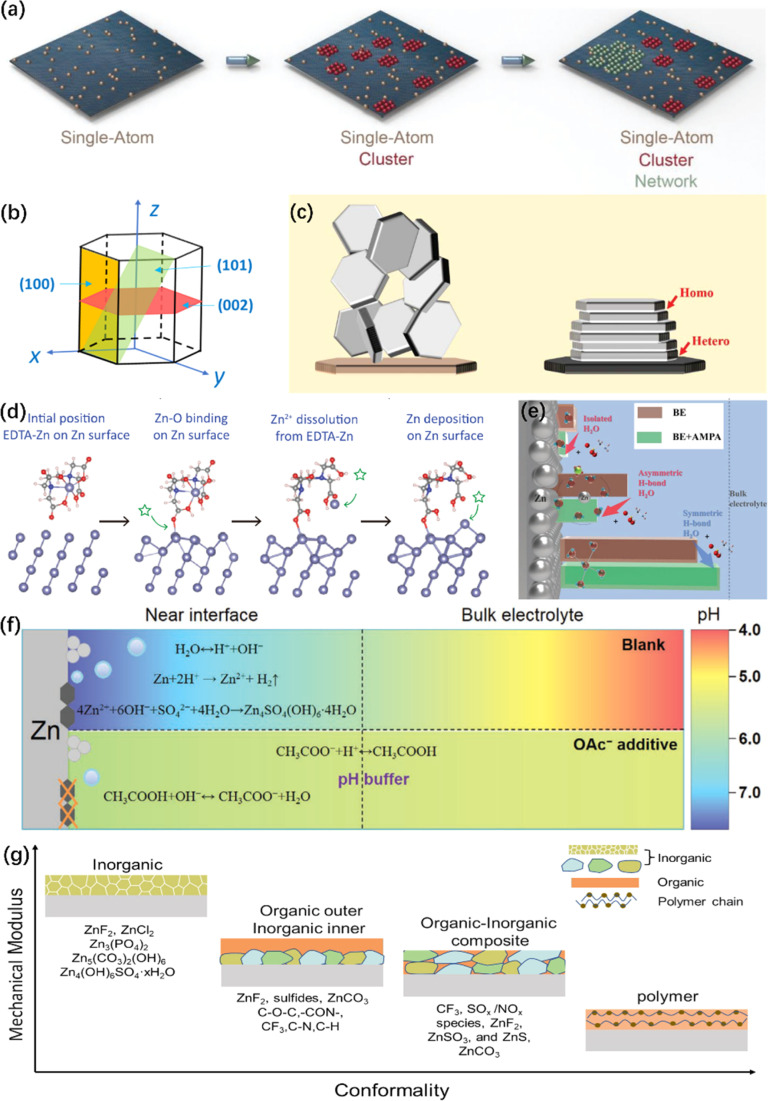
Illustration of electrolyte engineering for protecting ZMA. (a) Schematic illustration of the Zn deposition process.^50^ Copyright 2021, Wiley. (b) The illustration of the hexagonal close packed structure of Zn.^52^ Copyright 2023, Royal Society of Chemistry. (c) Scheme illustrating the design principle of epitaxial metal electrodeposition.^56^ Copyright 2019, AAAS. (d) Dynamic evolution of an EDTA–Zn molecule during Zn plating.^81^ Copyright 2022, Wiley. (e) Comparative schematic of interfacial water structures in different electrolytes.^91^ Copyright 2023, Wiley. (f) Schematic illustration of the pH evolution in different electrolytes and the interfacial pH buffer mechanism enabled by the OAc^−^ anion.^104^ Copyright 2022, Wiley. (g) Mechanical characteristics of different types of SEI layer.^25^ Copyright 2023, Wiley.

Furthermore, the incorporation of high valence cations additives, such as La^3+^ and Ce^3+^ ions, preferentially occupy the electron-rich active sites. This leads to Zn nucleation and growth taking place at relatively inert areas,^47^ weakening the repulsive force of electrical double layer (EDL)^48^ and guiding Zn nucleation towards gradual nucleation rather than instantaneous nucleation. Recently, Dai *et al.*^49^ introduced the concept of an “escort effect” of Ni^2+^ ion additives, which can deposit in advance and result in subsequent Zn plating *via* underpotential deposition. Regarding the relevant mechanisms, Xie *et al.*^50^ proposed a mesoporous Zn plating method, as illustrated in Fig. 3a. Hollow carbon spheres were chosen as the anode materials. Zn^2+^ was reduced to single-atom Zn on zincophilic sites in hollow carbon spheres, and then Zn clusters were formed by these single-atom Zn. These Zn clusters further expanded to form a mesoporous Zn network, leading to a uniform Zn plating layer. Apart from the electrolyte engineering, for more zincophilic site design, please consult ref. 51.

### 2.2. Regulating crystal orientation

Zn has a typical hexagonal close-packed (hcp) structure, as shown in Fig. 3b,^52^ which is characterized by crystal planes with high relative texture coefficients (RTCs), predominantly including the (002), (100) and (101) crystal planes. Among all the Zn crystal planes, the (002) crystal plane has a low surface energy. The (002)-preferred orientation generally provides active sites along the edge and induces paralleled stacked Zn plating on the ZMA.^53–55^ Based on this characteristic, Zheng *et al.*^56^ reported the epitaxial regulation concept of the Zn nucleation and growth, as illustrated in Fig. 3c. Furthermore, owing to its higher free energy of H adsorption and stripping energy of Zn, the (002) crystal plane could effectively inhibit HER and significantly avoid the side reaction.^35,55^ Consequently, a number of studies have recently designed to optimize the growth of the (002) crystal plane. The common methods of the (002) crystal plane modulation, such as surface texture design, surface coating, separator modification and current density control, have been systematically reviewed in ref. 57–60.

Apart from the abovementioned design and optimization, electrolyte additives also play an ingrained role during the plating process due to their specific adsorption effects. Sun *et al.*^61^ conducted research on a series of organic additives and concluded that using different additives in the plating process can modify the crystallographic properties and surface morphology of the ZMA. In this regard, the functions of these additives can be summarized as below.

(i) The additives, such as sodium 3,3′-dithiodipropane sulfonate,^52^ 1-butyl-3-methylimidazolium cation,^62^ and sorbitol,^63^ tend to adsorb on the other crystal planes except for (002). Therefore, more of the (002) crystal plane is exposed, inducing the preferential growth on the (002) crystal plane. For instance, the presence of I^−^ additives significantly elevates the growth rate along the (100) direction, thus resulting in the final exposure of the (002) crystal plane.^64^

(ii) Some additives, such as β-cyclodextrin, nicotinamide, and 1,4-dioxane, prefer to adsorb horizontally on the (002) crystal plane, thus modulating the nucleation and diffusion pathways of Zn^2+^ and guiding the orientated deposition along a direction parallel to the (002) crystal plane.^65–69^

(iii) Additives, such as l-cysteine,^70^ selectively etch the bulk Zn substrate, especially the Zn(101) crystal plane, and contribute to more dominant (002) crystal plane exposure.

Very recently, there have been divergent viewpoints. On the one hand, achieving a stable cycling performance with high capacity from (002)-textured zinc is challenging due to the significant lattice distortion and uneven distribution of the electric field.^71^ On the other hand, it is found that the (002) crystal plane is chemically unstable and prone to be corroded by water in aqueous electrolytes, leading to the formation of detrimental zinc hydroxide sulfate hydrate (ZHS).^72^ More importantly, it is unknown whether the above strategies are still effective when a higher depth of discharge is applied.

### 2.3. Modulating solvation structure

It is well known that the strong solvation of Zn^2+^ with H_2_O molecules exacerbates the electrochemical polarization and charge transfer resistance of Zn^2+^.^73^ Moreover, the strong interactions will lead to the HER of solvated water.^74^ A systematic summary and comprehensive introduction on the evolution of the solvation structure affected by electrolyte additives is presented in ref. 13. Typically, Zn^2+^ is solvated with six water molecules to form hydrated zinc ions, *i.e.*, [Zn(H_2_O)_6_]^2+^,^75^ leading to a high energy barrier of 289.3 kcal mol^−1^, and can be improved employing electrolyte additives.^61,76^ For instance, Xu *et al.*^77^ introduced silk fibroin (SF) as an electrolyte additive, and the [Zn(H_2_O)_4_(SF)]^2+^ solvation structure is formed. The desolvation process releases SF on the ZMA surface and *in situ* forms hydrostable and self-healable protective film. Yao *et al.* found that the triethylmethyl ammonium (TMA) cation can participate in the constitution of [Zn(SO_4_)_2_(TMA)_3_]^+^ structure in the electrolyte. The obtained solvated structure reduces the number of reactive H_2_O molecules and inhibits by-product formation.^78^ Moreover, methanol, as an antisolvent, can modulate the coordination structure of Zn^2+^.^79,80^ Based on the above-mentioned improvement of ZMA, it is necessary to analyze how additive molecules or cations inherently reconstruct the typical solvation structure, which could effectively improve the electrochemical performance. Yang *et al.* proposed a stability constant (*K*), which represents the equilibrium constant of the complexation reaction.^81^ This method is served as a universal standard to understand the anchoring strength between the additive ligands and solvated Zn^2+^, and the dynamic evolution of Zn plating process is shown in Fig. 3d. In addition, Shao's work^82^ reported that solvents with a higher donor number were favorable for stabilizing the ZMA–electrolyte interface as the donor number reflects the solvation ability between solvent molecules and Zn^2+^.

Apart from organic electrolyte additives or solvents, there are other categories of effective additives. Zhang *et al.*^83^ utilized a chloride salt with a bulky cation (1-ethyl-3-methylimidazolium chloride, EMImCl) to form an anion-type water-free solvation structure ZnCl_4_^2−^ in the electrolyte. They also designed an ammonium halide additive.^84^ I^−^ is acted as an electron donor and coordinates with Zn^2+^ to form the solvation structure ZnI(H_2_O)_5_^+^. The stability of the solvation structure is effectively enhanced, making it much easier to inhibit HER.

### 2.4. Weakening hydrogen bond network

The high activity of H_2_O molecules poses a threat at the ZMA-electrolyte interface.^85^ In addition, the original hydrogen bond network among H_2_O molecules, which is an important indicator of HER,^86^ can be easily destroyed by applying co-solvents or deep eutectic electrolyte.^87,88^ Moreover, a new hydrogen bonding network containing multifunctional groups can be constructed, which in turn inhibits the activity of the H_2_O molecule.^89^ For instance, xylitol additive inhibits HER, accelerates cation migration by expelling active H_2_O molecules, and facilitates the reconstruction of the hydrogen bond network.^90^ Recently, a study demonstrates that (aminomethyl)phosphonic acid (AMPA) promotes the formation of an ordered hydrogen bond network at the interfacial H_2_O, resulting in the inhibition of water-induced H_2_ production (Fig. 3e).^91^ The reconstruction of the hydrogen bond network enlarges the electrochemical stability voltage range and the operational temperature range.^92–94^ To validate the fundamental role of additives in the broken hydrogen bond network, a series of Lewis basic organic molecules with lone pair electrons were proposed.^95^ Due to the unique strong electronegativity of the lone pair of electrons, the tetrahedral structure of H_2_O molecules and their original hydrogen bond network were broken.

### 2.5. Maintaining proton concentration (pH)

According to the Pourbaix diagram, it is indicated that HER is a pH-dependent reaction and an inevitable thermodynamic process. The sources of HER on ZMA can be attributed to the following three reasons: the high reactivity of solvated H_2_O, the purity of ZMA and the irregular surface morphology.^96^ The occurrence of HER results in the release of more OH^−^, leading to an elevation in pH value and exacerbating the corrosion reactions on ZMA. For a thorough understanding, please consult the pH evolution law of the ZMA–electrolyte interface and its impact mechanism on the formation of Zn dendrites, as firstly revealed by Yang *et al.*^97^

Therefore, the pH value of the ZAM–electrolyte interface is crucial, and the additives will disrupt the pH value of the pristine electrolyte. As the amount of additive is increased, the pH value is either increased or decreased: the lower suitable pH of the electrolyte can diminish the formation of ZHS,^98,99^ and an appropriate increase in the electrolyte pH benefits the suppression of HER;^100,101^ moreover, an excessively high pH value (>5.47) would exacerbate corrosion.^102^ For instance, a specific quantity of Na_4_EDTA will trigger the formation of the ZHS precipitate in the electrolyte.^103^ Therefore, a pH buffer electrolyte additive is required to keep an appropriate pH value, such as acetic acid/acetate (HOAc/OAc^−^),^104,105^ pyridine/pyridinium,^106^ and imidazole/imidazolium.^107,108^ These electrolyte additives act as pH regulators to help maintain an appropriate acid–base balance during the charge/discharge process, ultimately mitigating the interfacial HER and corrosion reactions (as depicted in Fig. 3f).^104^ Interestingly, Zhang *et al.* presented a molecular switch strategy that utilizes the reversible structural changes of γ-butyrolactone (GBL) and γ-hydroxybutyrate (GHB) under varying pH levels.^109^ In addition, certain coating protection or double salt electrolyte can also serve as a pH buffer.^110–112^

### 2.6. Constructing solid electrolyte interphase (SEI) layer

The *in situ* construction of an Zn^2+^ conductive SEI layer that can be achieved through the reduction and decomposition of electrolyte, exhibiting enhanced durability and stability in providing protective effects. The presence of this *in situ* layer effectively impedes corrosion and promotes a homogeneous distribution of cations, attributable to its chemical bonding with the anode surface. Nevertheless, the establishment of a compact and enduring SEI layer in aqueous electrolytes faces a considerable obstacle due to the concurrent production of gas and inert byproducts during the decomposition of these electrolytes.^113^ Additional insights into the formation and composition of SEI can be ascertained in ref. 114, and Fig. 3g illustrates the mechanical characteristics of different types of SEI layer.^25^ Therefore, the introduction of an additional concept, referred to as the EDL, becomes imperative as it considerably impacts the chemical properties and morphology of the SEI through the arrangement of molecular and ionic assemblies in close proximity to the anode.^115^ However, numerous additives merely adsorb onto the ZMA surface to generate a EDL without undergoing subsequent reduction to form an SEI. In response to this issue, Huang *et al.* conducted a comprehensive evaluation of 15 organic additives and identified the pivotal determinant of these additives' protective effectiveness as their capacity to generate an SEI layer.^116^

Unlike the traditional organic electrolyte, ZMA suffer from severe surface corrosion and the formation of non-uniform ZHS and ZnO compounds. By introducing a small quantity of Zn(OH)_2_ additive into the ZnSO_4_ electrolyte^117^ or Na_2_SO_4_ additives into zinc trifluoromethanesulfonate (Zn(OTF)_2_) electrolytes,^118^ the conversion of the non-uniform ZHS into a homogeneous and durable SEI layer is achieved. Li *et al.* designed a low-concentration aqueous Zn(OTF)_2_–Zn(NO_3_)_2_ electrolyte. An insulating Zn_5_(OH)_8_(NO_3_)_2_·2H_2_O passivation layer was first formed, which then transformed into a more stable Zn-ion conductive Zn_5_(CO_3_)_2_(OH)_6_ layer and ZnF_2_-rich SEI layer in sequence.^119^ From the perspective of composition, prior investigations demonstrate that the SEI layers enriched in ZnF_2_,^120–122^ ZnS^123,124^ and zinc phosphate (Zn_3_(PO_4_)_2_) are propitious to enhance the stability of the ZMA–electrolyte interface.^125,126^ These constituents of the SEI layer are either derived from the decomposition products of additives or the reduction of anions promoted by additives.

Apart from inorganic additives, organic additives play an equally important protective role. For instance, the ZnCl_2_–H_2_O–DMSO electrolyte forms an SEI layer based on Zn_12_(SO_4_)_3_Cl_3_(OH)_15_ through the decomposition of solvated DMSO.^127^ On the one hand, organic additives can construct an anode-molecular interface.^128,129^ On the other hand, they can decompose directly to produce organic–inorganic SEI layers.^130,131^ In summary, additives containing nitrogen or sulfur functional groups can provide significant protection. However, the precise mechanism by which these active groups participate in the formation process of the SEI layer, especially when it involves the capture of certain intermediates, is poorly understood. Therefore, the advancement of theoretical simulations and *in situ* characterizations will contribute to uncovering the underlying mechanisms.

## 3. Theoretical simulations

Theoretical simulations have been widely conducted in literatures to investigate the mechanisms of reactions happening at the ZMA–electrolyte interface. Using theoretical simulations can considerably improve the understanding of the fundamental mechanisms.^132,133^ This section will specifically summarize the functions of various theoretical simulation methods in investigating the ZMA–electrolyte interface.

### 3.1. COMSOL simulations

**Fig. 4:**
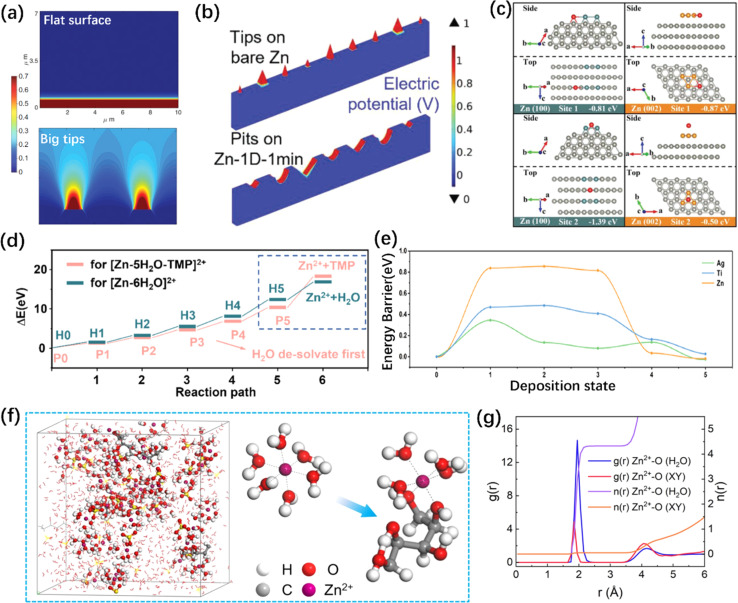
Illustration of theoretical simulations. (a) COMSOL simulation of Zn^2+^ diffusion and distribution with different conditions.^135^ Copyright 2019, Wiley. (b) Electric field distribution simulation results of ZMAs with different scratches.^136^ Copyright 2023, Wiley. (c) The adsorption energy of Zn at different sites on the (100) and (002) crystal planes.^55^ Copyright 2021, Wiley. (d) Comparison of the energy barrier required for normal and novel solvation structures to de-solvate through DFT calculation.^166^ Copyright 2023, Elsevier. (e) Energy profiles of Zn cluster at different deposition states.^169^ Copyright 2023, American Chemical Society. (f and g) Snapshot of the electrolyte containing xylitol additive (f) and its corresponding RDFs and CN (g) collected from MD simulations.^30^ Copyright 2023, Wiley.

As mentioned earlier, the stability of the ZMA–electrolyte interface can be influenced by interface roughness. An uneven interface can induce the “tip effect”, which depends on the intensity of the electric field and the Zn^2+^ concentration near the ZMA–electrolyte interface.^134^ As shown in Fig. 4a and b, COMSOL simulation indicated that electrons and ions tend to accumulate at the sharp tips on bare ZMA. Additionally, micron-scale concavities or pits on ZMA can also enhance the intensity of the electric field.^135,136^ The former worsens dendrite growth, while the latter is more conducive to enhancing the stability of the ZMA–electrolyte interface because it leads to a uniform electric field and Zn^2+^ distribution. Consequently, more researches on electrode structure design have been reported, such as 3D-scaffold anode^137,138^ and Zn@CuNW anode.^139–141^ Following this theory simulation, researchers discovered that an exaggerated electric field heterogeneity occurs when the bending radius is less than 5 mm.^142^ In terms of electrolyte engineering, COMSOL simulation can be applied to determine whether the interface electric field and Zn^2+^ flux are homogeneous by preferentially adsorbing functional groups on the ZMA surface.^143–145^ Furthermore, COMSOL simulations were roughly estimated to predict the surface morphology and the hydrogen diffusion flux.^122,146^

### 3.2. Density functional theory (DFT) calculations

It is generally accepted that DFT calculations are indispensable tools in numerous scientific and engineering disciplines, providing valuable guidance and explanations for experiments. In this section, the application of DFT calculations for the research of the ZMA–electrolyte interface is described as follows.

(i) Tuning the correlations, such as the adsorption energy of additive on ZMA–electrolyte interface, binding energy of additive with Zn^2+^, free energy of H adsorption^147,148^ and energy of the stripping process.^55,149^ Calculating and comparing the adsorption energy of zinc atoms on different crystal plane of ZMA helps identify the dominant zincophilic sites and provide reasons why additives can inhibit the growth of Zn dendrites.^44,50,150–153^ Moreover, the charge density distribution and surface electrostatic potential intuitively reflect the strong interaction of Zn^2+^ and precise active site for Zn^2+^ nucleation,^49,154,155^ respectively. As shown in Fig. 4c, DFT calculations show that the surface configuration^156^ or special Zn deposition sites^55^ can impact the adsorption energy of Zn atoms. Aside from zincophilic sites, lower diffusion energy barriers support the formation of a 2D deposition mode, leading to uniform and dendrite-free Zn deposition.^157,158^ Then, a unique zincophobic repulsion mechanism was proposed in the presence of an additive adsorption layer.^159,160^

In addition, the binding energy between different additives and Zn^2+^ is closely related to the solvation structure. In traditional electrolyte, Zn^2+^ solvated with six water molecules forms a hydrated zinc ions, resulting in a formation energy of −5.58 eV.^161^ By comparing the binding energy or desolvation barriers of different solvation structures, researchers can identify the most stable solvated structure and simulate the desolvation process.^162–165^ As shown in Fig. 4d, DFT calculations indicated that the desolvation process for additive molecule detachment can be considered as a rate-determining step. In this case, additive molecules tend to replace the active H_2_O molecules in the solvation structure, thereby driving the active H_2_O molecules away from the ZMA–electrolyte interface during the desolvation process. Thus, the HER caused by active H_2_O molecules is alleviated.^166^ Furthermore, Zhou *et al.* firstly investigated the effects of the molecular electrostatic polarity on the desolvation of hydrated Zn^2+^, which is used as a key factor to select a suitable additive to regulate reversible Zn plating/stripping chemistry.^167^

(ii) Evaluating the kinetics and stability of the Zn^2+^ deposition and diffusion on ZMA surface.^168^ Conventionally, it was believed that a low adsorption energy and rapid Zn^2+^ diffusion coefficient contribute to the fast and uniform nucleation and deposition of Zn hexagonal crystal.^143^ For instance, by comparing the 3D charge density variances and Gibbs free energy of the deposited Zn^2+^, as well as the energy of the Zn cluster on different matrixes (Fig. 4e), an accelerated reaction kinetics and uniform Zn deposition were implemented on Ag surfaces.^169^ Furthermore, to thoroughly investigate the mechanism of Zn dendrite formation induced by residual stress and lattice defects, the Gibbs free energy of Zn crystals under different stress and defect states was evaluated using DFT calculations.^170^

### 3.3. Molecular dynamics (MD) simulations

MD simulation can provide an in-depth electrolyte analysis, such as anion solvation structure,^171^ ionic conductivity,^172^ and spontaneous chemical reactivity.^173^ From the perspective of molecular dynamics, the evolution of the electrode–electrolyte interfaces can be simulated (Fig. 4f and g).^30^ The applications of MD simulations in the research of AZIBs to date can be summarized as follows. (1) Exploring the solvation structure of Zn^2+^ and its corresponding radial distribution functions (RDFs) and coordination number (CN).^174–176^ (2) Elucidating the ionic diffusion behaviors in the electrolyte or the SEI layer.^141,177–179^ (3) Estimating the hydrogen bond network and activity of H_2_O.^80,92,180^ (4) Revealing the effects of additive molecules on the separator or ZMA.^129,181^

Therefore, MD simulations can help understand the changes in the electrolyte during a proposed system in a visualized way. For instance, an electrochemically and thermally stable Zn_5_(OH)_6_(CO_3_)_2_-containing SEI layer was successfully achieved by the decomposition of *N*,*N*-dimethylformamide (DMF).^182^ The corresponding MD simulation indicates that most of the Zn^2+^ migration occur in the Zn_5_(OH)_6_(CO_3_)_2_ phase along the [010] lattice plane. Moreover, the concentration-dependent effects in an electrolyte-containing propylene glycol (PG) additive were studied by the MD simulation, indicating that a self-assembled mediated film formation occurs at a low concentration.^183^ Recently, Yang *et al.* proposed a bulk-phase reconstructed ZMA with abundant zincophilic sites, which considerably improved the resistance to dendrite growth and side reactions even after deep stripping. In the case, MD simulations were employed to analyze the nitrogen bombardment process at different nitrogen species energies, substrate temperature, and substrate vacancy concentrations to guide the acquisition of a bulk-phase reconstructed ZMA.^184^

## 4. *In situ* characterization

Theoretical simulations provide valuable insights into the protective mechanisms of electrolyte engineering strategies and guide their further development. Furthermore, experimental evidence, obtained through *in situ* characterizations, is also indispensable for a direct understanding of the mechanisms. In this section, we summarize the applications of various novel *in situ* characterizations that have been employed to investigate the ZMA–electrolyte interface, such as *in situ* visualizing characterizations, *in situ* spectroscopy/mass spectrometry characterizations, *in situ* physicochemical characterizations and other advanced characterizations. For a schematic representation of the relevant *in situ* characterizations and more detailed information about the *in situ* battery structure, please consult the references of 185–187.

### 4.1. *In situ* visualizing characterization

#### 4.1.1. *In situ* optical microscopy (OM)

**Fig. 5:**
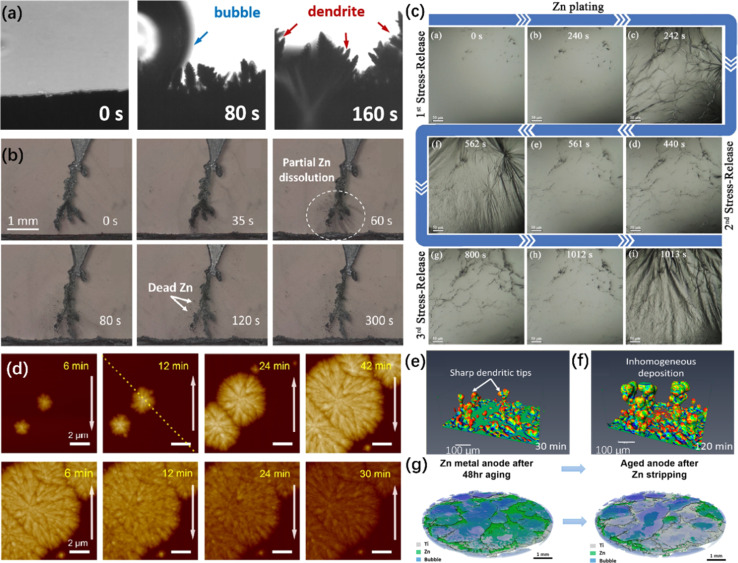
*In situ* visualizing characterizations of ZMA. (a) *In situ* OM of the ZMAs in traditional electrolytes during the plating process.^42^ Copyright 2023, Royal Society of Chemistry. (b) The dissolution of Zn dendrites and the production of dead Zn under the subsequent stripping process.^190^ Copyright 2023, American Chemical Society. (c) The stress-generation-release phenomena on the anode at different plating stages.^197^ Copyright 2022, Wiley. (d) *In situ* AFM images of nucleation and early growth and dissolution of Zn.^204^ Copyright 2021, American Chemical Society. (e and f) Selection of region of interest (ROI) demonstrating the distribution of the mean curvature of the Zn dendrite as a function of time at (e) 30 and (f) 120 min.^190^ Copyright 2023, American Chemical Society. (g) 3D rendered images from the anode of a pressured Zn/Ti battery scanned *in situ* X-CT.^212^ Copyright 2023, Elsevier.

Similar to Li-ion batteries,^188,189^ it is essential to monitor the morphology and structure evolution of the ZMA in real-time. ZMA protection strategies have been well studied using *in situ* OM to investigate the differences of dendrite growth under different conditions. Without any protection strategies, irregular dendrites and gas bubbles form on the ZMA surface during the plating process, which is due to the inhomogeneous Zn nucleation and severe HER in traditional electrolyte (Fig. 5a).^42^ Moreover, during the stripping process, the partial dissolution of Zn could be observed (Fig. 5b),^190^ which leads to the formation of “dead Zn” and consequently low coulombic efficiency (CE).^191,192^ A dendrite-free ZMA can be achieved after the application of protection strategies.^134,193–195^ Through the *in situ* OM observation, pits are formed after the first stripping process. These pits exacerbate the subsequent growth of dendrites. In contrast, pre-deposited ZMA exhibits a more uniform morphology in subsequent cycles.^196^

Except for the above cross-section observation, the surface-section perspective is also extensively utilized to observe the interfacial flatness and nucleation sites.^192^ For instance, a stress-generation-release phenomena on the coating layers during the Zn plating process was directly observed, as shown in Fig. 5c. In detail, a large number of Zn grains emerge on the ZMA surface and soon vanish in a flash. As the plating time increases, significant fluctuations and wrinkles appear due to the huge stress exceeding the threshold coating layer strain. Immediately afterwards, the ZMA surface gradually recovers to a flat state due to the groove deposition model.^197^

*In situ* OM is also applied to reveal the effects of current density on Zn plating. Generally, higher current density is supposed to result in more Zn dendrite formation on the ZMA surface,^198^ which is considered as a guidance to control the plating behaviors and crystal orientation of Zn deposition by tuning the current densities.^199^ However, Yu *et al.* clearly demonstrated that the *in situ* OM technique used for observing ZMA is not a sufficiently reliable characterization for describing the HER and dendrite growth process due to the significant randomness of various deposition morphologies in different regions.^27^

#### 4.1.2. *In situ* atomic force microscopy (AFM)

*In situ* AFM is capable of providing surface roughness, Young's modulus, and 3D surface configuration images and is widely used in the field of energy storage devices.^200–202^ For AZIBs, the plating and stripping processes of Zn could be monitored by *in situ* AFM.^203^Fig. 5d illustrates the surface morphology evolution on ZMA within a single Zn plating/stripping process. During the plating process, two nascent nuclei emerge and then gradually turn into micron-sized particles through radial expansion. During the stripping process, Zn dendrites are homogeneously dissolved identified by a color change from dark yellow to light yellow.^204^ Besides, AFM images are used to monitor Zn dendrite formation at different current densities and depth of discharge. At a very early stage, the size of the dendrite increases while a higher current density is applied. Besides, the formation of Zn dendrites is accelerated with a higher depth of discharge.^135^

In addition, AFM is also used to support the effects of surface modification. For example, Chen *et al.* polished the Zn foil using sand papers with a proper grit size, which demonstrate a stable cycling performance. AFM images showed that Zn deposition mainly happens in the concaves generated by sand papers and proves that smaller grit size contributes to flatter anode surface, *i.e.*, dendrite free surface.^192^ Zhang *et al.* performed *in situ* AFM to reveal the plating patterns in different electrolytes. As a result, a dot-distributed Zn nucleation mechanism was observed in traditional ZnSO_4_ electrolyte. However, an anion-type water-free Zn^2+^ solvation structure electrolyte leads to plane-distributed Zn nucleation.^83^ Keist *et al.* investigated the evolution of Zn deposition morphology using *in situ* AFM and concluded that uniformly distributed Zn nucleus gradually become larger and thicker in a layer-by-layer growth mode, leading to the smooth surface and improved cycling performance in an imidazolium-based ionic liquid electrolyte.^155,205^ Moreover, Wang *et al.* presented that a smoother hexagonal surface and pyramid-like structures with sharp edges were observed in a concentrated electrolyte through *in situ* AFM images, whereas a rough and undulating topography was observed in a dilute electrolyte.^206^ Despite the use of *in situ* AFM for the direct observation of the surface topology of ZMA during the first plating stage, using *in situ* AFM to monitor the formation of SEI layers or EDL remains underexplored.

#### 4.1.3. *In situ* X-ray computed tomography (X-CT)

X-ray computed tomography (X-CT)^207,208^ has been employed to achieve the 4D observation of the Zn plating/stripping process. As shown in Fig. 5e and f, after a statistical quantification of the mean curvature, the 3D rendering image for the entire domain can be drawn, and different colors represent different compositions.^190,208^ Therefore, X-CT is suitable for studying the 3D structure of ZMA, such as the surface morphology of the cycled Zn@3DCu_3_ anode^209^ and the spatial distribution of ZnO in porous ZMA.^210^ Meanwhile, Pu *et al.* employed the *in situ* X-CT technique to demonstrate plated Zn and Zn substrates without disassembling the battery. The obtained results further indicate that the epitaxial Zn does not possess interior defects after the plating.^211^ They also demonstrated that the gas bubbles generated on the Zn surface can physically deactivate Zn in the following plating/stripping processes, which is monitored by the *in situ* X-CT technique (Fig. 5g).^212^ In addition, the X-CT technique can verify the detailed and homogeneous Zn plating behavior after constructing a protective SEI layer or employing additives.^213^ For instance, Zhang *et al.* demonstrated that the homogeneous Zn plating behavior can be achieved on the hydrophobic–zincophilic SEI layer because a relatively flat surface has been detected by *in situ* X-CT,^214^ while extensive “dead Zn” was shown in *in situ* X-CT images when using additive-free electrolyte.^215^ Although the above mentioned examples demonstrate the potentials of the X-CT technique in promoting a deep understanding of the protective mechanism, several challenges still need to be overcome, including expanding the scanning range and improving the accuracy and resolution of the detected area.^216,217^

#### 4.1.4. *In situ* electron microscopy

**Fig. 6:**
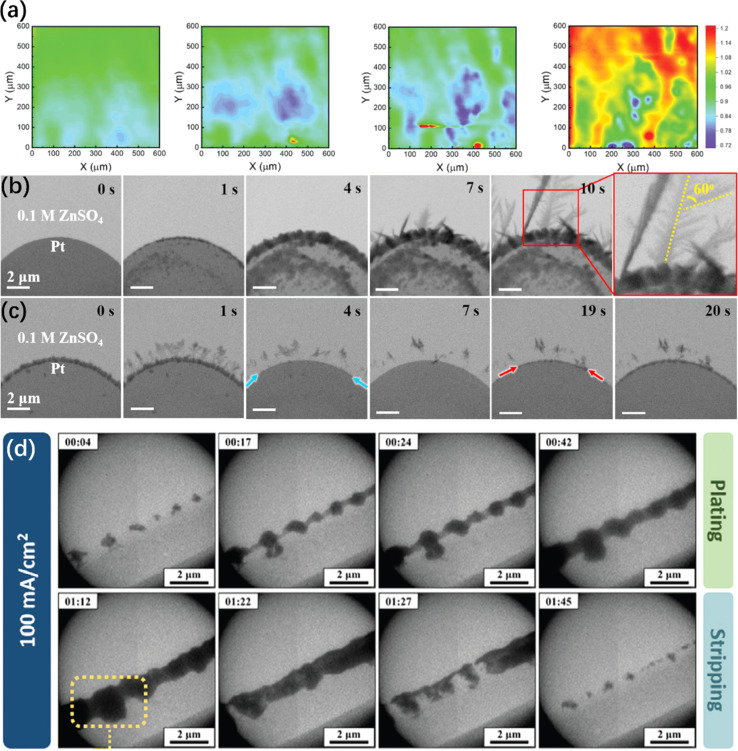
Other *in situ* electron microscopy characterizations of ZMA. (a) *In situ* SECM feedback imaging of Zn electrodeposition process on freshly polished Zn substrates.^218^ Copyright 2023, Wiley. (b and c) *In situ* TEM images of the zinc plating/stripping process on Pt electrode.^219^ Copyright 2021, Elsevier. (d) *In situ* electrochemical LC-TEM imaging of Zn plating/stripping with LiCl additive.^220^ Copyright 2024, Wiley.

To monitor the local chemical reactions on the interface with higher resolution, electron microscopy, such as *in situ* scanning electrochemical microscopy (SECM) and *in situ* transmission electron microscopy (TEM), is employed. As shown in Fig. 6a, vertical oriented patterns are observed on the polished ZMA through *in situ* SECM. In contrast, an SEI layer can guide the horizontal accumulation of thin Zn sheets at the early stage of Zn deposition to suppress the growth of dendrites.^218^ As indicated by *in situ* TEM, particles and needle-like precipitates are formed on the Pt electrode during the plating process (Fig. 6b), occurring before 4 s and after 7 s, respectively. The dendritic branch angle is approximately 60°. During the stripping process (Fig. 6c), the particles near the Pt surface is more likely to be stripped away than the dendrite tips and the detached Zn dendrite is a potential risk for “dead Zn”.^219^ Very recently, an electrostatic shielding effect mechanism has been further confirmed *via in situ* liquid-cell TEM, which is close to the practical AZIBs batteries.^220^ In this case, as shown in Fig. 6d, LiCl will induce preferential secondary nucleation on the previously deposited Zn flakes along the (002) plane, resulting in the formation of stacked zinc flakes.

### 4.2. *In situ* spectroscopy/mass spectrometry characterizations

#### 4.2.1. *In situ* Raman spectroscopy

**Fig. 7:**
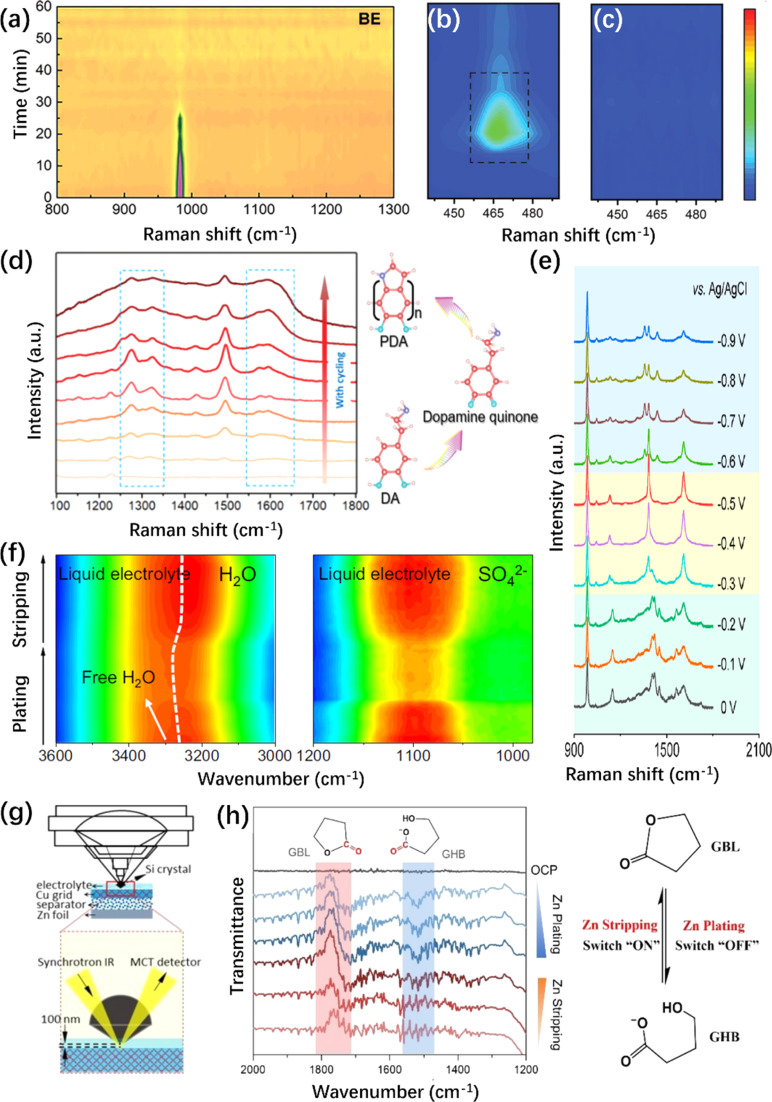
*In situ* Raman/FTIR spectroscopic characterizations of ZMA. (a) *In situ* Raman spectrum intensity map of (a) SO_4_^2−^ anion in pristine electrolyte.^224^ Copyright 2022, Wiley. (b and c) *In situ* Raman spectrum of Zn(OH)_4_^2−^ species on (b) Cu@Zn and (c) MGA@Zn anode.^227^ Copyright 2022, Wiley. (d) *In situ* Raman spectra of DA additive and its representative polymerization procedure for PDA.^231^ Copyright 2021, Royal Society of Chemistry. (e) *In situ* Raman spectra of the PI-COF electrode during the charging process.^232^ Copyright 2020, American Chemical Society. (f) *In situ* ATR-FTIR spectra of the O–H band and SO_4_^2−^ anion vibration.^237^ Copyright 2023, Springer Nature. (g) Illustration of piezo-controlled macro-ATR monitoring of the interfacial area of the electrode.^238^ Copyright 2023, Wiley. (h) *In situ* ATR-FTIR spectra of conformation changes between GBL and GHB.^109^ Copyright 2023, Wiley.

In contrast to the visualization research discussed in the above section, *in situ* Raman spectroscopy can provide a deeper understanding of the vibration information of molecules. So far, *in situ* Raman spectroscopy has been widely used to analyze the corrosion behavior of ZMA.^221,222^ For instance, the gradually emerging O–H stretching vibration of ZHS at 3633 cm^−1^ indicates the existence of irreversible corrosion reaction.^223^ The spectrum intensity of the SO_4_^2−^ peak at 980 cm^−1^ becomes weakened during the plating process (Fig. 7a), suggesting that SO_4_^2−^ was consumed due to ZHS formation.^224^ Moreover, the side reaction HER generated more OH^−^, which further accelerates the formation of Zn(OH)_4_^2−^ species, hindering the subsequent transport of Zn^2+^. Therefore, a Raman peak located at 465 cm^−1^ in Fig. 7b, assigned to the Zn(OH)_4_^2−^ species, could be observed in the traditional electrolyte.^225^ The corrosion behavior can be suppressed by modifying ZMA *via* an oriented freezing process or thermal infusion strategy^226,227^ because the Zn(OH)_4_^2−^ peak at 465 cm^−1^ has disappeared, as shown in Fig. 7c.

Referring to the complexity of the EDL or the SEI layer on the ZMA, *in situ* Raman is an effective tool to monitor the *in situ* formation of the EDL and SEI layer.^228^ For instance, with the increase in the deposition time, the vibration peaks of the 3-(1-methylimidazole)propanesulfonate (ImS) additive gradually increased and would not change upon cycling; it is supposed that ImS can adsorb on ZMA to form EDL without undergoing chemical decomposition.^229^ In addition, the gradual polymerization of the monomers to form the corresponding polymer is recorded by *in situ* Raman during cycling.^230^ As shown in Fig. 7d, the intermediates of *in situ* polymerization are detected, which would lead to the successful construction of a robust polydopamine layer on the ZMA eventually. This *in situ* polymeric SEI possesses abundant functional groups and outstanding hydrophilicity for regulating Zn nucleation to achieve dendrite-free Zn plating.^231^

In the case of artificially-constructed ZMAs, *in situ* Raman spectroscopy also plays a significant role in exploring the mechanism of Zn^2+^ storage. For instance, the vibration mode of a 2D polyarylimide covalent organic framework (PI–COF) indicates its reversible conversion during cycling (Fig. 7e).^232^ Furthermore, *in situ* Raman can be used to identify the preferential Zn^2+^ nucleation sites, such as the diacetylene bonds in the graphdiyne nanowalls^233^ and the O and OH terminal on the Ti_3_C_2_T_*x*_ MXene.^234^

#### 4.2.2. *In situ* Fourier transform infrared spectroscopy (FTIR)

*In situ* FTIR techniques can also support the study of ZMA by identifying the formation of new species and monitoring the decomposition of the electrolyte. Generally, the attenuated total reflection (ATR) configuration is used in *in situ* FTIR experiments, especially in battery systems. Amaral *et al.* have summarized a plenty of *in situ* FTIR configurations and their corresponding significant advancements.^235^ As for the ZMA–electrolyte interface, *in situ* FTIR has been widely employed to monitor molecular dynamics during plating/stripping, particularly the migration of Zn^2+^ and the desolvation process.^91,236^ As shown in Fig. 7f, during the plating process, the peak of the O–H band from the H_2_O molecule exhibits a blueshift, and the peak intensity of SO_4_^2−^ becomes weakened. These could be explained as a repulsion between H_2_O molecules and SO_4_^2−^ anions resulting from the desolvation of Zn^2+^.^237^ Liu *et al.* employed *in situ* synchrotron FTIR characterization to monitor the effects of electrolyte additives on the hydrogen bond network, and the corresponding configurations are illustrated in Fig. 7g.^238^ With the introduction of the additive, a redshift of O–H is monitored together with increased peak intensity, which means a new hydrogen bond network between H_2_O and additives is established and the general water derived hydrogen bond network is destroyed.^39,239^ Moreover, *in situ* FTIR enables the direct observation of the reduction behavior of additive molecules, such as dimethyl methylphosphonate (DMMP) and *N*,*N*-dimethylformamidium trifluoromethanesulfonate (DOTf).^238,239^ Zhang *et al.* reported γ-butyrolactone (GBL) as the electrolyte additive to stabilize the pH value at the ZMA–electrolyte interface.^109^ ATR-FTIR spectroscopy was used to monitor the transformation between GBL and γ-hydroxybutyrate (GHB) to unveil the mechanism for the adjustable pH in the battery system. Specifically, the reversible C=O vibration during Zn plating/stripping has been identified, as shown in Fig. 7h.

#### 4.2.3. *In situ* X-ray diffraction (XRD)

**Fig. 8:**
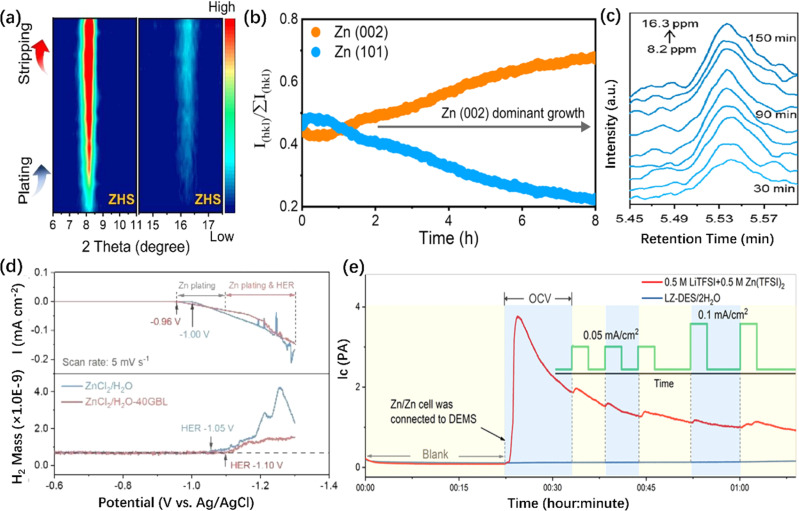
*In situ* X-ray diffraction/mass spectrometry characterizations of ZMA. (a) *In situ* XRD pattern of the ZMA without/with the HZTO protective layer.^223^ Copyright 2023, Wiley. (b) *In situ* XRD pattern and corresponding RTCs of the MXene array ZMA.^234^ Copyright 2023, Springer Nature. (c) *In situ* EC-GC of pristine ZMA during the plating/stripping process.^250^ Copyright 2023 American Chemical Society. (d) *In situ* DEMS characterizations of the electrolyte without/with GBL additive.^109^ Copyright 2023, Wiley. (e) *In situ* DEMS characterization of the electrolyte with 0.5 M LiTFSI + 0.5 M Zn(TFSI)_2_ and LZ-DES/2H_2_O electrolyte.^252^ Copyright 2019, Elsevier.

*In situ* XRD is mainly used to investigate the phase transformation of active materials.^240,241^ More details of the advanced *in situ* XRD characterizations about the cathodes for AZIBs are discussed in ref. 242–245. As for ZMA, *in situ* XRD technique can identify byproducts formation and measure crystal plane changes during the plating/stripping process. For instance, ZHS is a main byproduct while using ZnSO_4_ based electrolyte and can be easily identified in the XRD data. Moreover, the characteristic peaks of ZHS are irreversible (Fig. 8a).^223,246^ Secondly, the oriented deposition behavior of Zn is also a hot topic, which could be monitored by collecting the corresponding relative texture coefficients (RTCs) values of all the crystal planes by *in situ* XRD. For example, the preferred oriented Zn deposition on ZMA could be identified by the XRD result as the (002) peak gradually becomes stronger. Generally, the preferred Zn(002) deposition means a horizontal stacked Zn plate on ZMA, ensuring a smooth ZMA during cycling (Fig. 8b).^234,247,248^ Furthermore, *in situ* XRD can be used to study the evolution of ZMA structure and its reversibility, contributing to the analysis of “dead Zn” formation.^227,249^

#### 4.2.4. *In situ* differential electrochemical mass spectrometry (DEMS) and gas chromatography (GC)

Generally, the amount of H_2_ produced by the modified or protected ZMA is much less than that produced by pristine ZMA, known as HER inhibition. Bubble formation is hardly seen in the modified system, but it does not mean that the HER is completely eliminated. Therefore, *in situ* electrochemical gas chromatography (EC-GC) or Differential Electrochemical Mass Spectrometry (DEMS) are employed. For EC-GC, the amount of gas production can be quantified over a specific time period (typically 15 minutes), as shown in Fig. 8c.^74,103,250^ Based on DEMS, continuous cumulative gas production can be detected.^160,251^ Furthermore, the combination of linear sweep voltammetry and DEMS can accurately distinguish the potentials for Zn plating and HER (Fig. 8d).^109^Fig. 8e demonstrates the H_2_ evolution during the open-circuit voltage (OCV) state.^252–254^ Also, the presence of plenty fluctuation peaks indicates the HER and released bubbles.^255^ Briefly, these *in situ* EC-GC or DEMS methods are primarily utilized for evaluating the H_2_ evolution rate and electrolyte stability.^256^

### 4.3. *In situ* physicochemical characterizations

Intrinsic properties, such as impedance, mass, and electrolyte conditions, are also crucial factors for AZIBs. These intrinsic properties affect the electrochemical performance, interface reactions, and electrolyte stability. Investigating the changes in intrinsic properties holds great significance in improving the protection strategies, *i.e.*, more stable ZMA–electrolyte interface, less side reaction, and higher safety.

#### 4.3.1. *In situ* electrochemical impedance spectroscopy (EIS)

**Fig. 9:**
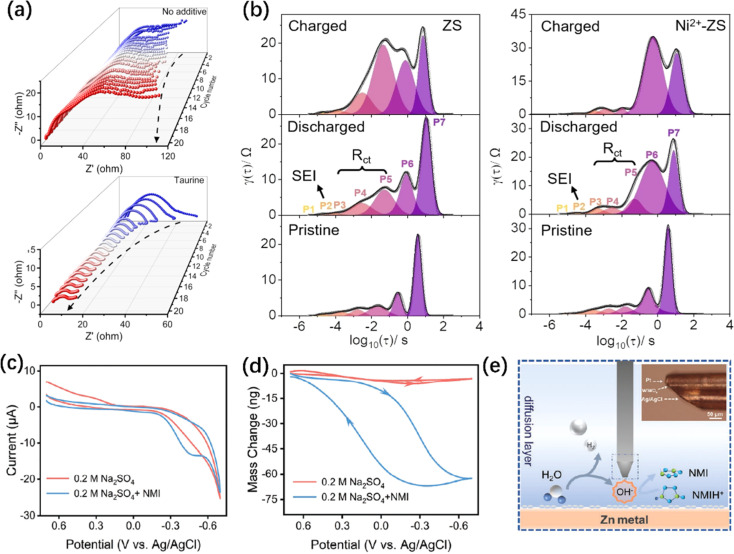
*In situ* physicochemical characterizations of ZMA. (a) *In situ* EIS of symmetrical batteries without/with taurine additive electrolyte.^14^ Copyright 2023, Wiley. (b) The distribution of relaxation times (DRT) plot of the *in situ* EIS data in ZS and Ni^2+^-ZS electrolyte.^49^ Copyright 2023, Wiley. (c and d) Cyclic voltammograms (CV) curves (c) and corresponding mass change (d) obtained from EQCM test.^107^ Copyright 2023, Wiley. (e) Diagram of UME detecting pH changes (inset is a microscopy image of pH UME).^107^ Copyright 2023, Wiley.

Electrochemical impedance spectroscopy (EIS) is an essential method to study reaction kinetics at the ZMA-electrolyte interface and the evolution of the SEI layer.^257,258^ As shown in Fig. 9a, the *in situ* EIS results demonstrate that the interface resistance of the pristine electrolyte gradually increases. On the other hand, the interface impedance in taurine electrolyte is more stable even after a drastic reduction.^14,259^ Such a result reflects the fast interface transport kinetics, originating from the elimination of the generation of ZSH.^260–262^ In detail, during the plating process, the interface impedance decreases slightly, indicating that the newly deposited Zn nanoarrays expose more active sites.^263^ For a more comprehensive analysis, Dai *et al.* recorded the *in situ* EIS spectra and the performed distribution of relaxation time (DRT) analysis (Fig. 9b).^49^ The interfacial impedance (P3–P5) in the ZnSO_4_ electrolyte increases significantly after the first cycle of plating/stripping, while that in the Ni^2+^–ZnSO_4_ electrolyte remains relatively constant, corresponding to a significant decrease in the ZHS. In addition, *in situ* EIS can provide information about the surface roughness and “soft shorts” of ZMA during cycling.^264,265^

#### 4.3.2. *In situ* electrochemical quartz crystal microbalance (EQCM)

The electrochemical quartz crystal microbalance (EQCM), as a gravimetric analysis tool, establishes a relationship between the mass change and charge transfer of the active materials. Consequently, it can analyze the specific adsorption of additive, in-depth plating/stripping mechanisms, and formation of SEI.^266,267^ Zhang *et al.* carried out EQCM to assess the absorption behavior of *N*-methylimidazole (NMI) additive on ZMA (Fig. 9c and d). There is a significant mass change in the electrolyte containing NMI additive when the potential changes, which is attributed to the adsorption of NMIH^+^ on the interface at low potential and the desorption of NMIH^+^ at high potential.^107^ In addition, EQCM records a negligible mass change resulting from the adsorption and desorption of H atoms and H_2_O in the pristine electrolyte.^268^

The plating/stripping process of Zn^2+^ is generally believed as a two-electron reaction. However, Agrisuelas *et al.* conducted EQCM studies and characterized the presence of the Zn^+^ intermediate, suggesting that the plating/stripping of Zn^2+^ undergoes two consecutive single-electron transfer steps.^269^ In addition, EQCM is used to identify the side reactions such as the formation of byproduct. The mass change of ZMA is detected by EQCM during immersion in electrolyte with/without dissolved oxygen. Much faster mass increment is recorded due to the growth of byproducts induced by dissolved oxygen during the first resting state.^270^ Another example is the detection of SEI formation. Significant unbalanced data of coulombic loss and gravimetric mass change is observed in the non-aqueous Zn(TFSI)_2_ electrolyte due to the SEI formation process in the first three cycles. However, in the aqueous electrolyte, no significant loss of charge occurs, resulting in nearly 100% CE.^271^

#### 4.3.3. *In situ* proton concentration (pH)

Moreover, proton concentration in the electrolyte plays an important role in AZIBs, showing up as the pH value at the ZMA–electrolyte interface. Generally, because Zn^2+^ plating/stripping and HER process share an overlap potential range, which means that within the battery operation voltage range, HER is inevitably triggered.^272,273^ Symmetric batteries with conventional electrolyte suffer from a gradually increased pH environment, indicating the continuously occurring HER reaction.^101,106,112,274^ Therefore, researchers applied an electrolyte additive to maintain the stability of the pH value, which has been described in Section 2.5.^14,101,275^ Recently, Zhang *et al.*^107^ used a ternary pH ultra-microelectrode (UME, Fig. 9e) to detect the pH value by controlling the distance between the probe and ZMA to distinguish the pH evolution between the bulk and diffusion layers. The results indicate that the OH^−^ species produced by HER will not diffuse into the bulk electrolyte because of the strong interactions between OH^−^ and Zn^2+^ at the interface. However, there is no generally accepted suitable pH values for AZIBs because both low pH (2.8)^102^ and high pH (5.14)^104^ have been reported to favor ZMA stability.

### 4.4. Minority and advanced characterizations

**Fig. 10:**
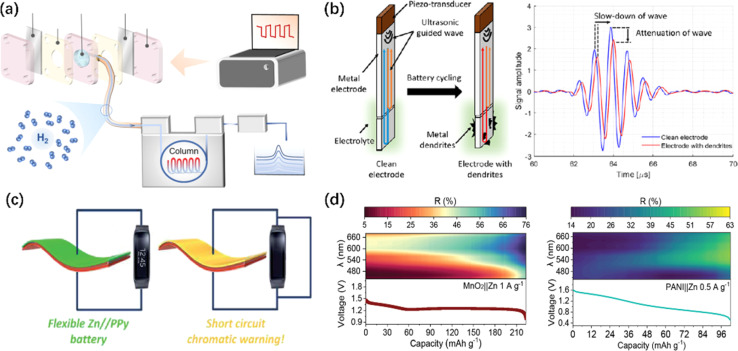
Minority and advanced characterizations of ZMA. (a) Schematic of the configuration used for observing H_2_ evolution *in situ*.^276^ Copyright 2024, Springer Nature. (b) Schematic of a transducer-waveguide assembly and ultrasonic signals on a waveguide before (blue) and after (red) the cycling.^277^ Copyright 2022, Elsevier. (c) Schematics of a chromatic battery with a short circuit warning function.^279^ Copyright 2018, Royal Society of Chemistry. (d) Scheme illustrating the process of smartphone multispectral imaging reconstruction and usage for *in situ* energy storage monitoring.^280^ Copyright 2023, Wiley.

Apart from the commonly used *in situ* characterizations mentioned above, some relatively rare but innovative strategies and characterizations have been used in AZIBs. As shown in Fig. 10a, the generation of bubble footprints can be observed on the surface of cycled ZMA, while *in situ* GC collects the bubbles and separates the hydrogen gas for analyzing whether the HER reaction is inhibited.^276^ The results indicate that uneven zinc deposition is more likely to occur around the bubbles, leading to the easier formation of dendrites. Moreover, a novel ultrasonic-guided wave technique was introduced (Fig. 10b) by Zhang *et al.* With the beginning of Zn plating, the velocity and attenuation of ultrasound change. These changes are related to the formation of dendritic structures on the ZMA–electrolyte interface. As dendrites grow, the attenuation of ultrasound increases, and the velocity decreases. The reason for its attenuation is that Zn dendrites increase the scattering and absorption of energy. Conversely, during the stripping process, the wave velocity increases.^277^

In addition, significant advancements and applications have been achieved in the integrated characterizations of full-battery. For instance, Zhang *et al.* reported a flexible pressure sensor composed of a solid-state ZIB, which can be used for accurate and continuous human pulse and limb movement detection. This ZIB-type pressure sensor can be used as both an energy storage device and an excellent pressure sensor, which effectively convert mechanical signals into electrical signals and output a stable high/low frequency response signal depending on the various interface resistance and isolation layer resistance.^278^ Similarly, a flexible and electrochromic AZIBs has been reported by Wang *et al.* Once a short circuit occurs in the battery, the voltage rapidly drops to 0 V, and the polypyrrole (PPy, as cathode) layer quickly transitions from the oxidized state to the reduced state, causing the battery's color to change from black (normal operation state) to bright yellow (short circuit state).^279^ Sun *et al.* proposed a smartphone computing multispectral imaging reconstruction (MSIRC) strategy to unlock the ion storage behaviors in cathode materials. As indicated in Fig. 10d, by leveraging the relative reflectance values at specific wavelengths, a “layer to layer” Zn^2+^ storage mechanism and excessive Zn^2+^ insertion was identified.^280^ Researchers used a smartphone to perform the *in situ* analysis of aqueous batteries through a transparent monitoring window. The authors extracted the optical characteristics of the materials using computational reconstruction methods, thereby enabling the monitoring of the chemical ion storage and real-time capacity changes of the materials. Most recently, artificial intelligence has become more and more popular. Further investigations may take the advantage of this powerful tool to guide the research design, help understand complex mechanisms and kinetics, and accelerate the acquisition of experimental and computational data.

## 5. Summary and outlook

In conclusion, AZIBs possess the advantages of high safety, low cost, and eco-friendliness and are one of the most promising energy storage and conversion devices for the applications in smart grid. Zn is considered as the most ideal anode material but encounters challenges such as Zn dendrites, HER, and corrosion. This review summarizes the theoretical simulations and multiscale *in situ* characterization for understanding the protective mechanism of the ZMA–electrolyte interface. Nonetheless, researches on AZIBs are still at its early stages, and there are many critical scientific issues and underlying mechanisms waiting to be disclosed, as detailed below.

**Fig. 11:**
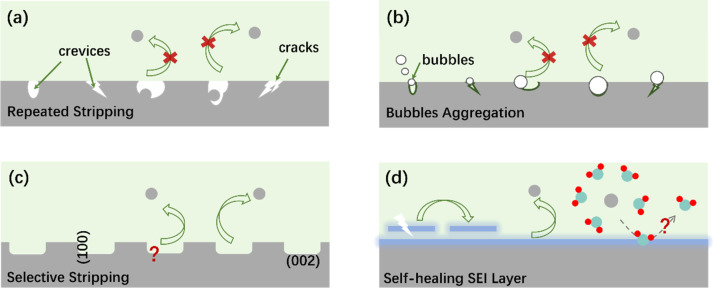
Scientific issues and prospects for the plating/stripping process of ZMAs.

(i) The stripping mechanisms receive less attention, despite the fact that the stripping process is the first step of ZMA in a full battery.^184,281^ As shown in Fig. 11a, the uneven stripping process leads to the formation of voids and/or cracks on ZMA, exposing highly active surface. The volume changes of ZMA after repeated cycles lead to the formation of interspace/crevices between the modified layer and ZMA. The modified layer is thus easier to separate from the ZMA and loses its protective effect.

(ii) Inhibiting the bubble formation is challenging since it is difficult to fully control the HER. As shown in Fig. 11b, the gradual aggregation of bubbles physically isolates some Zn with the electrolyte, leading to the capacity attenuation of ZMA. However, this issue is normally neglected since excessive Zn is applied.

(iii) Similar to the preferred oriented Zn plating, is it possible to achieve selective stripping processes? For instance, selectively stripping Zn(101) and exposing more favorable Zn(002) (Fig. 11c) would in turn promote the subsequent plating process along the (002) crystal surface.

(iv) Is the EDL completely covered with adsorption-type additives? If not, is there still a significant amount of solvated H_2_O that can directly contact ZMA? Therefore, it is crucial to distinguish the state of local environment from the near interface region to the bulk electrolyte.

(v) It is challenging to fabricate a functional SEI with high performance. For instance, when constructing the SEI layer, it is necessary to consider its thickness, mechanical strength, and self-healing ability. As shown in Fig. 11d, excellent self-healing ability is beneficial for extending the battery life. Moreover, it is important to investigate and understand how the composition and microstructure of SEI influence the desolvation and diffusion coefficient of Zn^2+^.

These issues severely affect the development of AZIBs in practical applications. It is important for researchers to understand the relationships among crystal structure, material composition, reaction mechanism, reaction kinetics, and electrochemical performance through advanced tools such as theoretical simulations and *in situ* characterizations mentioned in this review. Besides, when it comes to *in situ* characterizations, researchers need to pay more attention to the reliability and validity of the required data and quality for practical applications.

(i) Developing and integrating a variety of *in situ* characterizations that can operate closer to actual operating conditions are essential for a more accurate understanding of the dynamic changes at the ZMA–electrolyte interface.

(ii) Improving the temporal and spatial resolution of *in situ* characterizations is crucial to rapidly capture the electrochemical processes occurring and subtle structural changes, which are essential for understanding the operation mechanisms and optimizing the performance of AZIBs.

## Author contributions

Z. L. conceptualized and reviewed the articles and drafted the manuscript. X. Z. and Z. L. surveyed the relevant literature and analyzed the table data. Y. H. and Z. H. revised and provided general oversight. All the authors have approved the final version of the manuscript.

## Conflicts of interest

The authors declare no conflict of interest.

## Supplementary Material
